# Utilizing Suture Anchors in Addition to Screws for Treatment of Tibial Tubercle Avulsion Fracture: A Case Report

**DOI:** 10.7759/cureus.56363

**Published:** 2024-03-18

**Authors:** Anna Matsuo, Kazuya Kaneda, Kohei Michifuri, Teppei Hayashi, Hideo Morioka

**Affiliations:** 1 Orthopaedic Surgery, National Hospital Organization Tokyo Medical Center, Tokyo, JPN; 2 Orthopaedic Surgery, Keio University School of Medicine, Tokyo, JPN

**Keywords:** orif, avulsion fracture, extensor mechanism, pullout strength, suture anchor, surgical technique, tibial tubercle fracture

## Abstract

Tibial tubercle avulsion fractures are relatively uncommon fractures commonly seen in adolescent males. The treatment goal is to restore the extensor mechanism and to repair the articular surface. Although previous surgical techniques have been mainly screws or tension band wiring, there is a certain consensus on this. However, the choice of these surgical techniques largely depends on the surgeon. In our case, we utilized a suture anchor distal to the cannulated screw. This enabled us to use a smaller screw and cover the screw head completely with the patellar tendon. Therefore, this can be an advantage in lowering the incidence of device irritation. Given the successful outcome of our technique, we may consider applying suture anchors more frequently in tibial tubercle avulsion fractures in the future.

## Introduction

A tibial tubercle avulsion fracture is a type of fracture that occurs when a sudden contraction of the knee extension mechanism puts stress on the tibia tubercle, causing it to avulse. Tibial tubercle avulsion fractures account for 0.4% to 2.7% of pediatric fractures and less than 1% of all epiphyseal fractures [1]. A notable prevalence has been observed in male adolescents, with an average age of 14.6 years. This is attributable to the fact that the closure of the physis occurs between 10 and 15 years old in females and 11 and 17 years old in males [1].

The goal of treatment is to retrieve the extensor mechanism and repair the articular surface. When the fracture is dislocated or involves the articular surface, open reduction and internal fixation (ORIF) are indicated [2]. Various fixation techniques using screws, tension band wiring, or sutures have been reported; however, there is no golden standard for this fracture [2-6]. Here, we report a case of displaced tibial tubercle avulsion fracture successfully managed with a small screw combined with a suture anchor, and we provide a review of previously reported techniques.

## Case presentation

**Figure 1:**
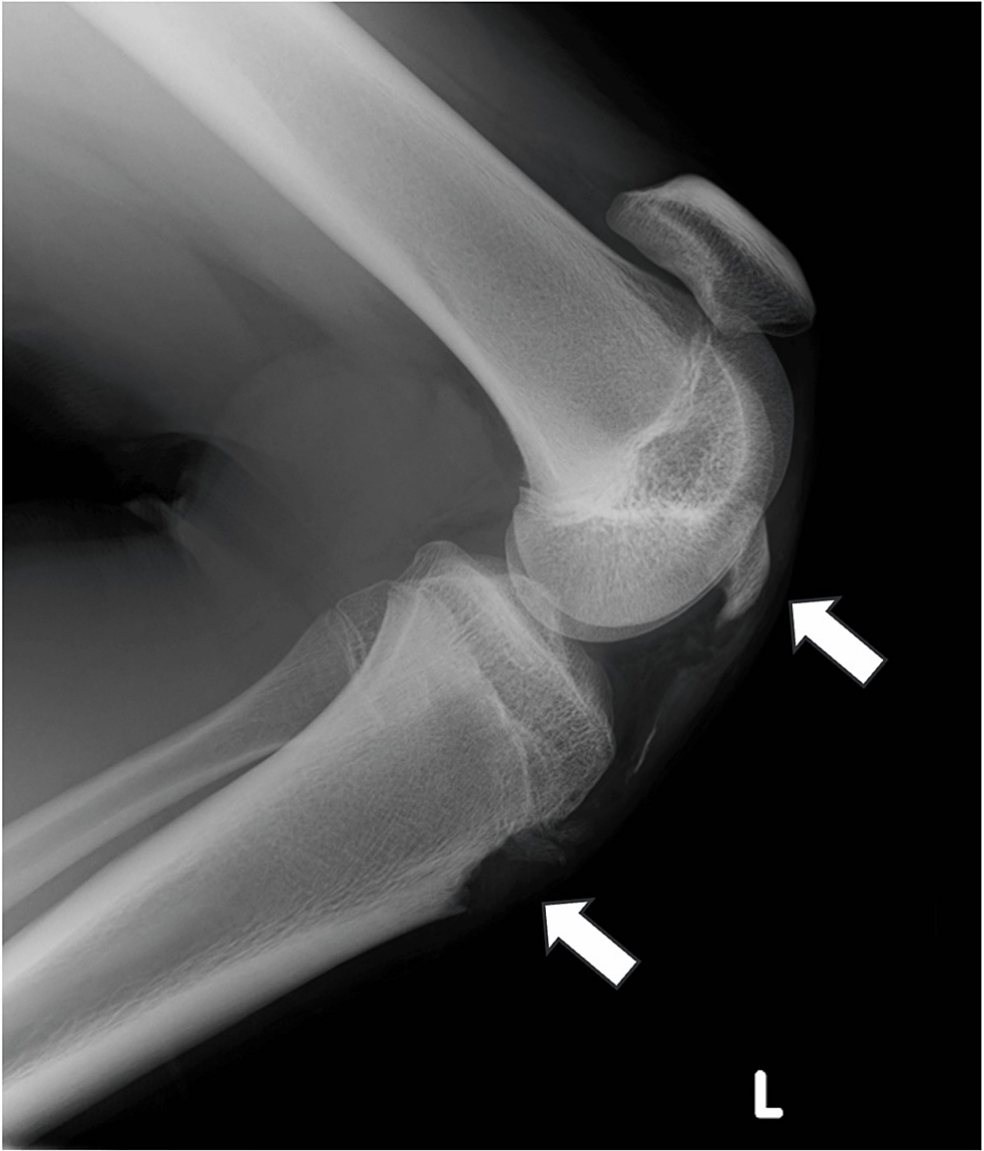
Preoperative radiograph shows Ogden type ⅠB tibial tubercle fracture.

**Figure 2:**
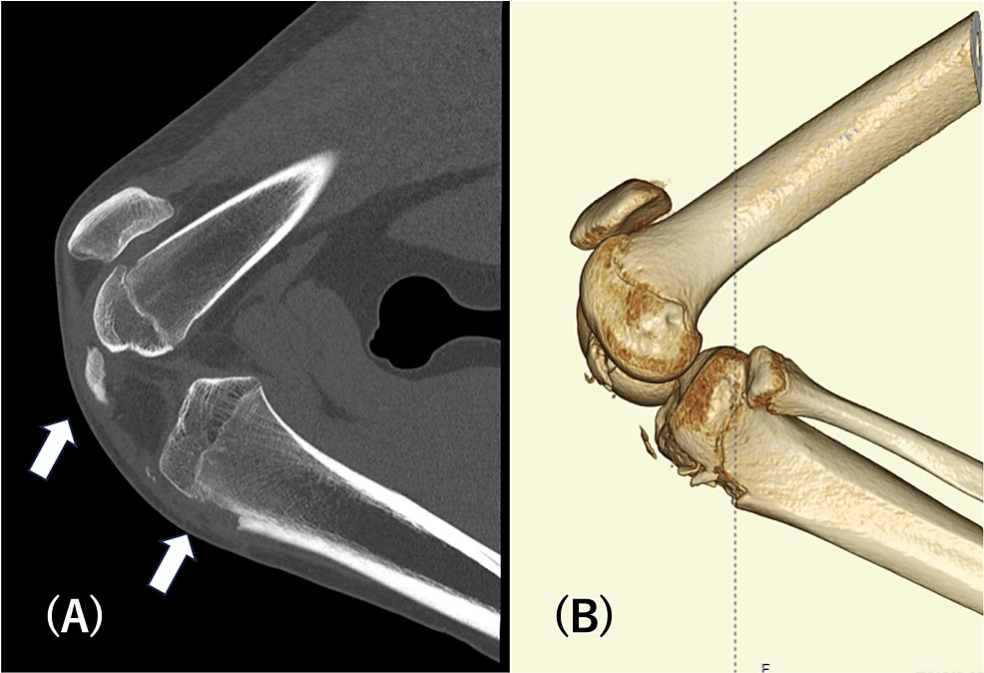
Preoperative computed tomography images show no sign of intra-articular involvement.

A 13-year-old adolescent male presented to the emergency department with acute pain in his left knee while practicing hurdles as a part of school sports. Upon examination, the patient was hemodynamically stable but exhibited notable swelling and tenderness with difficulty in active extension (range of motion 30-100 degrees). Plain radiographs and computed tomography scans showed a tibial tubercle avulsion fracture with no intra-articular involvement, classified as Ogden type ⅠB (Figures 1-2) [7]. Since the fragment was dislocated and the extensor mechanism was damaged, ORIF was performed the following day.

**Figure 3:**
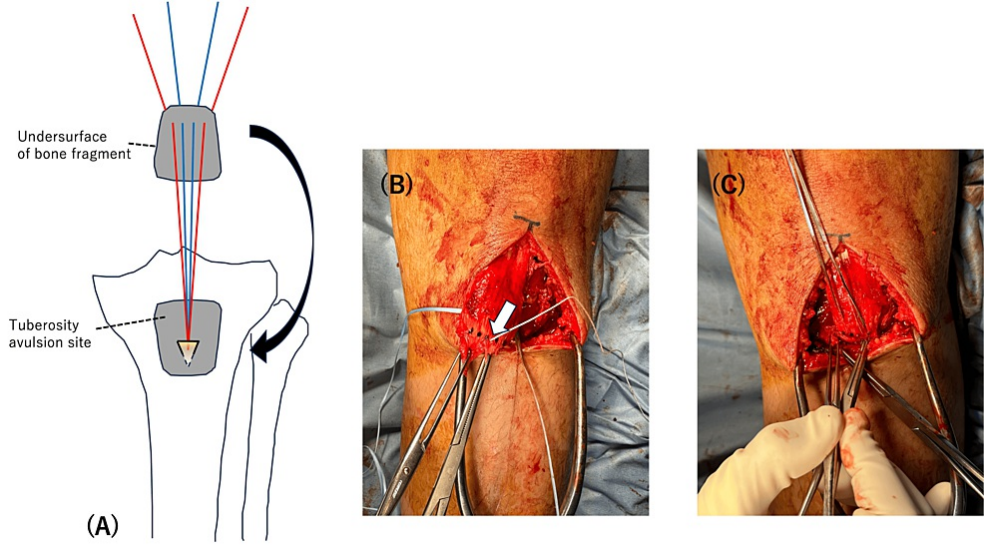
Surgical procedure of the suture anchor. (A) Sutures were pulled through the bone fragment; (B) the dots show where to puncture; (C) pulling the sutures to make sure the fragment is repositioned in the right place.

**Figure 4:**
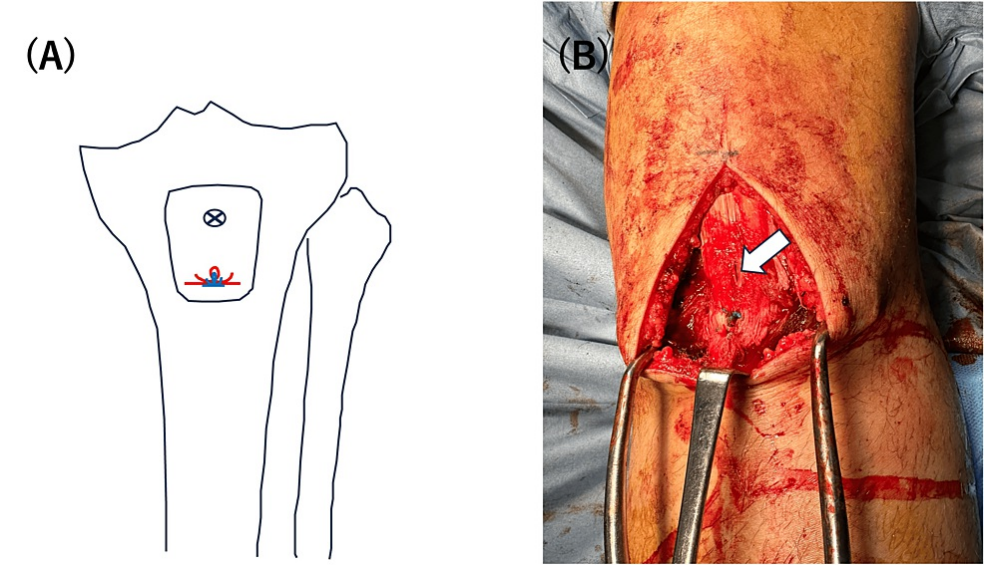
Surgical procedure of the screw. (A) After the sutures are tied, a screw was placed proximally; (B) white arrow shows the screw covered with the patellar tendon.

**Figure 5:**
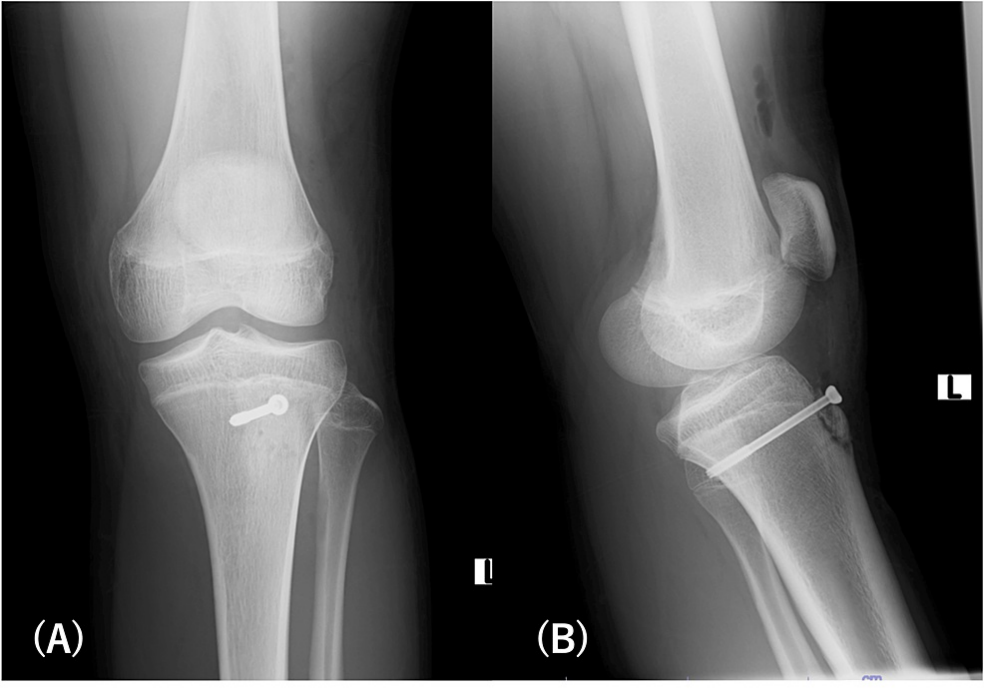
Postoperative radiographs.

The surgical procedure was performed under general anesthesia with the patient in the supine position. The position of the patella in the unaffected knee was assessed by fluoroscopy for comparison during a surgical procedure. An anterior midline incision, measuring 10 cm in length, was made, extending from the proximal pole of the avulsed fragment to the tibial tubercle. The avulsed fragment was successfully repositioned without identifying any ruptures in the patellar tendon. A 4.5-mm suture anchor was placed on the distal third of the fracture bed. Four sutures from the anchor were passed from posterior to anterior through the bone fragment aligned in a horizontal way (Figure 3). The medial and lateral sutures were tied, respectively (Figure 4A). A 4.0-mm partially threaded cannulated screw without a washer was inserted into the proximal third of the bone fragment over the fragment toward the posterior tibial cortex, ensuring no interference with the sutures (Figure 5). The screw was inserted by splitting the patellar tendon, and after insertion, the head of the screw could be covered by the patellar tendon (Figure 4B). To prevent excessive reduction of the bone fragments, the knee joint was positioned in 30° flexion as opposed to extension. This was done with reference to the position of the patella on the unaffected knee. The periosteum was repaired with absorbable sutures to support the fixation.

**Figure 6:**
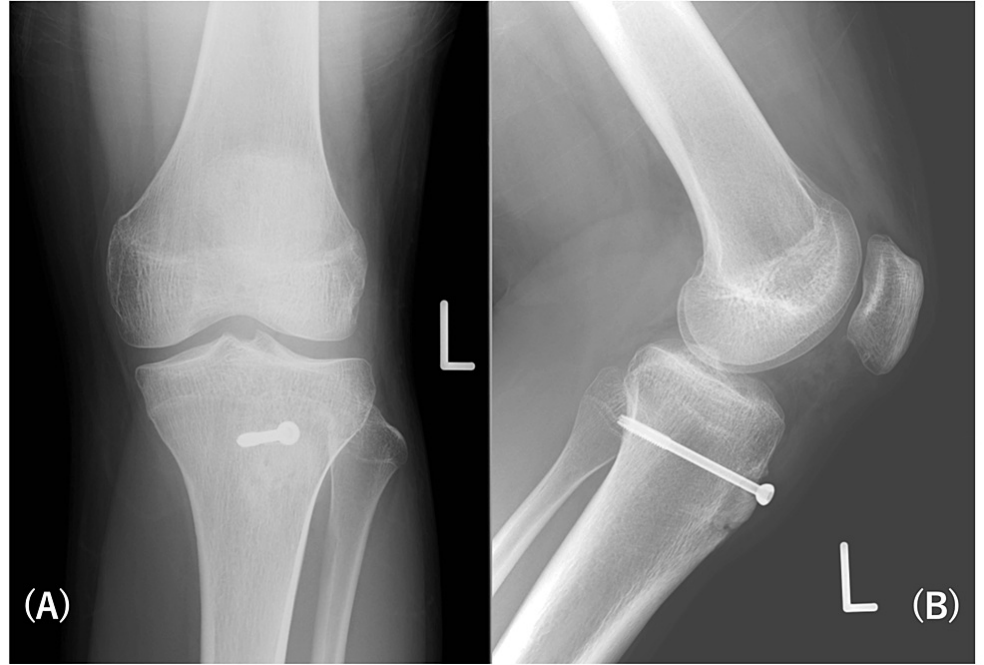
Six months postoperative radiograph.

The post-operative plan included a brace in full extension for four weeks and non-weight bearing for four weeks. Weight bearing was allowed by one-third of body weight every two weeks to reach full body weight at eight weeks post-op. Radiographic union was observed after three months (Figure 6). At six months post-op, the patient had returned to his original sports level without limitation in range of motion (0-130°) or any device irritation and was satisfied with the outcome.

## Discussion

Tibial tubercle fracture is a relatively rare condition that typically occurs in adolescent males (males/females, 10:1) [1]. The paramount objective of management is to repair the extensor mechanism and the articular surface. The severity and classification of the fracture determine the treatment. There are two types of treatment: non-operative and operative. For minimally displaced fractures (<2 mm), immobilization with a long leg cast in extension for three to four weeks can serve as treatment. Fractures that are displaced (>2 mm) or have intra-articular involvement need surgical intervention. In terms of classification, fractures more severe than OgdenⅠB are to be considered surgery. In cases that may involve the articular surface, further screening with an MRI, arthroscopy, or arthrotomy is required to evaluate associated injuries such as meniscus tears [8,9].

Pretell-Mazzini et al. reported that 88% of tibial tubercle fractures undergo surgical treatment, and within these surgical cases, ORIF was performed in 98% [8]. Techniques using tension bands or screws have been predominantly favored. However, a consensus has not yet been reached, and the procedure depends mostly on the surgeon’s preference.

Polakoff et al. reported on 12 cases treated by tension band wiring [3]. All fractures exhibited proper healing, and this method has been used for a long period of time. Tension band wiring can also be combined with screws. Park et al. reported a technique for tension band wiring with screws. In their case, instead of utilizing Kirshner wires to make a figure of eight, they used the patellar tendon and the insertion site of the tibial tubercle to wrap the wire. The authors indicate that tension band wiring enables an early range of motion [4].

Screw fixation is another common technique. Rickert et al. recommend the use of 4.5 or 6.5-mm cannulated, partially threaded screws. In this study, all patients resumed full activity with complete radiographic healing with a 10% to 20% complication rate, including partial physical arrest, decreased range of motion, painful implants, etc. [5]. Another serious potential complication associated with this technique would be injuring the popliteal neurovasculature during anterior-posterior drilling [10]. Although bicortical screws facilitate more rigid fixation, unicortical screws can avoid neurovascular complications. Arkader et al. compared the treatment outcomes among 51, 13, and 23 patients with the use of unicortical, mixed, and bicortical screws, respectively, and found no significant differences among the screw types [11]. In 2023, Zukotynski et al. reported on 71 patients who were treated with unicortical screws, and all had adequate healing and returned to full activities [12]. Given the rarity of this complication, it is inconclusive as to which screw is superior [6].

In our case, we used one 4.0-mm partially threaded cannulated screw and a 4.5-mm suture anchor. As previously noted, 4.5-mm screws are typically used for smaller fragments and 6.5-mm screws for larger fragments [5]. Fully threaded knotless anchors are reported to have sufficient pullout strength [13]. By utilizing an anchor, a smaller screw became available. In addition, our technique was unique in that the screw was placed proximally so that it could be completely covered by the patellar tendon. No screw heads were exposed prior to wound closure. Symptomatic hardware has been reported in the literature to be about 6%, and the present method is thought to be an advantage in lowering the complication, which may require device removal [8,10,14]. There are various other ways to use suture anchors. For example, Howarth et al. used suture anchors in the epiphysis to repair the meniscal-articular relationship in Ogden type Ⅲ or Ⅴ fractures [2]. Lee et al. reported a case of tibial tubercle avulsion fracture in an adult in which the main fragment was too small for either screw fixation or tension band wiring. Therefore, a suture bridge technique was solely performed to successfully secure the tibial tuberosity [15].

Regardless of the surgical technique, the reports were mostly successful. Kalifis et al. reported fracture union in 99.8% of cases, regardless of the fracture type and treatment [14]. Furthermore, the overall complication rate is low, and the outcomes are considered excellent [6,16]. Our case also resulted in a successful outcome. Our technique to place a smaller screw proximally and a suture anchor distally could potentially lead to fewer complications associated with device irritation. Therefore, we advocate for this technique.

## Conclusions

The outcome of performing suture anchor techniques in addition to screws was satisfactory in this case of a tibial tubercle fracture. Even though screws and tension band techniques are still part of conventional treatment, the surgical procedure mainly depends on the surgeon. Suture anchors are proven to have adequate pullout strength, which can serve as a substitute or reinforcement for the other techniques. Through our case, we suggest that the application of suture anchors in this way may be considered more.
